# ECOG-ACRIN E6293: Phase II Study of Raltitrexed in Advanced Colorectal Cancer

**DOI:** 10.1093/oncolo/oyaf378

**Published:** 2026-02-20

**Authors:** Chengwei Peng, Paul Catalano, John Zalcberg, Ann D Thor, Louis M Weiner, Janardan D Khandekar, Deirdre Cohen, Eric L Weinshel, Peter J O’Dwyer, Al Bowen Benson

**Affiliations:** Division of Hematology/Oncology, Department of Medicine, Robert H. Lurie Comprehensive Cancer Center Northwestern University, Chicago, IL, 60611, United States; Department of Biostatistics, Harvard T H Chan School of Public Health, Cambridge, MA, 02115, United States; Department of Medical Oncology, Alfred Health, Melbourne, 3004, Australia; Research Program, Monash School of Public Health, Melbourne, 3004, Australia; Department of Pathology, University of Colorado, Aurora, CO, 80045, United States; Department of Oncology, MedStar Georgetown University Hospital, Washington, DC, 20057, United States; Department of Medicine, Endeavor Health, Evanston, IL, 60201, United States; Department of Hematology/Oncology, Mount Sinai Hospital, New York, NY, 10029, United States; Department of Hematology/Oncology, Fairview-Southdale Hospital, Edina, MN, 55435, United States; Department of Hematology/Oncology, University of Pennsylvania, Philadelphia, PA, 19104, United States; Division of Hematology/Oncology, Department of Medicine, Robert H. Lurie Comprehensive Cancer Center Northwestern University, Chicago, IL, 60611, United States

**Keywords:** raltitrexed, colorectal cancer, antifolate inhibition

## Abstract

**Background:**

Inhibition of thymidylate synthase (TS) is a common mechanism in the treatment of colorectal cancer (CRC). 5-Fluorouracil is an indirect inhibitor of TS that is commonly used in CRC treatment regimens. Raltitrexed is a direct TS inhibitor that was hypothesized to have better efficacy and toxicity in CRC due to its specific inhibition. To test this, a phase II ECOG-ACRIN trial testing raltitrexed was conducted.

**Methods:**

This trial took place from December 1995 to December 1998. Advanced CRC patients were enrolled into three strata: (1) No prior treatment (2) One prior line of 5-FU-based regimen without leucovorin (3) One prior line of 5-FU-based regimen with leucovorin. Raltitrexed 3 mg/m^2^ was given every 3 weeks. A two-stage design with pre-specified ORR was utilized. Primary endpoints were ORR, toxicity, and prognostic value of TS expression by immunohistochemistry. Secondary endpoints were mPFS and mOS.

**Results:**

One hundred one patients were enrolled. ORR, mPFS, and mOS (months) by stratum were:

1. 5-FU Naïve: 3%, 2.1 (95% CI 1.4, 2.7), 14.5 (95% CI 8.0, 19.9),

2. 5-FU regimen/no leucovorin: 4.2%, 2.6 (95% CI 1.4, 3.5), 12.5 (95% CI 5.0, 17.0), and

3. 5-FU regimen/leucovorin: 3.3%, 1.7 (95% CI 1.4, 2.3), 7.3 (95% CI 4.9, 14.3).

Based on low ORR, the trial did not advance to the second stage. One PR occurred in the high TS expression group, none in low TS group.

**Conclusion:**

In this phase II trial, raltitrexed did not show significant response rates in patients with advanced CRC. Due to limited ORR of raltitrexed, value of TS expression as a biomarker was inconclusive. No new safety signals for raltitrexed were demonstrated.

Lessons Learned:

- Raltitrexed did not demonstrate significant activity in colorectal cancer.
- Further analysis of targeting antimetabolite pathways and biomarkers of response is necessary.

## Trial information

**Table:** 

Trial information	
**Disease**	Colorectal cancer
**Stage of disease/Treatment**	IV
**Prior therapy**	At most 1 prior line of treatment
**Type of study**	Phase II ECOG-ACRIN Study (ECOG-ACRIN E6293)
**Primary endpoints**	Overall response rate, toxicity, predictive value of thymidylate synthase expression on response
**Secondary endpoints**	Overall survival and progression-free survival
**Additional details of endpoints or study design** **This trial took place from December 1995 to December 1998. The trial was originally designed with the following three strata: (1) No prior chemotherapy (2) Prior 5-FU-based regimens, but no leucovorin (3) Prior 5-FU-based regimens that included leucovorin. This was amended to the following criteria: (1) No prior treatment (2) One prior line of 5-FU-based regimen without leucovorin (3) One prior line of 5-FU-based regimen with leucovorin.** **The statistical design was a two-stage design with response rates evaluated separately in the three strata. A true response rate of 30% was considered active for patients in stratum 1, while response rates of 15% were considered promising for patients who had received prior treatments (strata 2 and 3).** **In stratum 1, 24 patients were to be initially entered, assuming that at least 22 would be eligible. If there were at least four responses among the 22 eligible patients, an additional 24 patients would have been entered, of whom 22 would have been assumed eligible. If at least nine responses were observed among the 44 eligible patients, then the treatment would have been considered promising for that group.** **For the previously treated patients (strata 2 and 3), 28 patients were to be initially entered into each stratum, assuming that at least 26 would be eligible. If there were at least two responses among the 26 eligible patients in each stratum, an additional 20 patients would have been entered, of whom 18 would have been assumed eligible. If at least four responses were observed among the 44 eligible patients in each stratum, then the treatment would have been considered promising for that subgroup of patients.** **Thymidylate synthase IHC staining levels was incorporated as a biomarker of response. IHC staining was graded as 0, 1+, 2+, and 3+, with comparison made between 0/1+ and 2/3+ staining.**	

## Drug information

**Table:** 

Drug information	
**Generic/Working name**	Raltitrexed
**Company name**	AstraZeneca
**Drug Type**	Thymidylate synthase inhibitor
**Drug class**	Antifolate metabolism
**Dose**	3.0 mg/m2
**Route**	IV
**Schedule of administration every 21 days**	

## Patient characteristics

**Table:** 

		Stratum	
Patient characteristic	1 (*N* = 34)	2 (*N* = 27)	3 (*N* = 40)
**Age**			
** Mean (range)**	62 (45-82)	59 (36-82)	62 (29-82)
**Sex, *n* (%)**			
** Male**	19 (56)	20 (74)	27 (68)
** Female**	15 (44)	7 (26)	13 (32)
**Race, *n* (%)**			
** White**	27 (79)	25 (93)	34 (85)
** Black**	4 (12)	1 (4)	4 (10)
** Other**	3 (9)	1 (4)	2 (5)
**ECOG, *n* (%)**			
** 0**	20 (59)	11 (41)	19 (48)
** 1**	14 (41)	16 (59)	21 (53)
**Tumor location, *n* (%)**			
** Right**	12 (35)	7 (26)	16 (40)
** Left**	22 (65)	17 (63)	19 (48)
** Unknown**	0	3 (11)	5 (13)
**Sites of metastases, *n* (%)**			
** Liver**	28 (82)	19 (70)	34 (85)
** Lung**	10 (29)	9 (33)	15 (38)
** Peritoneum**	3 (9)	2 (7)	5 (13)

## Primary assessment method

**Table:** 

Primary assessment method	
**Title**	Overall response rate, PFS, OS
**Number of patients screened**	106
**Number of patients enrolled**	101
**Number of patients evaluable for toxicity**	100
**Number of patients evaluated for efficacy**	100
**Evaluation method**	Response rate as determined by ECOG-ACRIN Solid Tumor Response Criteria from imaging
**Outcome Notes: 101 patients were enrolled onto the trial; however, 87 patients met the final strata criteria. Response rates for the revised strata (*n* = 87) are listed in Table 1 and for the overall population (*n* = 100) in Table 2. PFS and OS data are listed in Table 3 which is for the overall population with the original strata inclusion criteria. Survival curves are shown in Figure 1 (PFS) and Figure 2 (OS).**	

Secondary assessment method	
**Title**	Thymidylate Synthase (TS) IHC
**Number of patients evaluated for IHC**	51
**Evaluation method**	IHC staining
**Outcome Notes: Limited number of patients available for IHC evaluation and due to lack of response rates, unable to conclude if TS IHC can be used as biomarker, results in Table 4.**	

## Serious adverse events (SAEs)

**Table:** 

Life-threatening (Grade 4) toxicities		
Case number	Toxicity type	Stratum
**62061**	Granulocytopenia	1
**62076**	Liver	1
**62026**	Liver	2
**62054**	Leukopenia	2
**62054**	Thrombocytopenia	2
**62054**	Diarrhea	2
**62054**	Granulocytopenia	2
**62003**	Granulocytopenia	3
**62006**	Granulocytopenia	3
**62020**	Diarrhea	3
**62023**	Liver	3
**62032**	Leukopenia	3
**62036**	Leukopenia	3
**62036**	Diarrhea	3
**62036**	Granulocytopenia	3
**62088**	Thrombocytopenia	3
**62088**	Liver	3
**62097**	Vomiting	3

## General toxicity profile

**Table:** 

Stratum	1 (*N* = 34)			2 (*N* = 26)			3 (*N* = 40)		
**Grade**	1,2	3	4	1,2	3	4	1,2	3	4
**Leukopenia**	8	1	0	6	0	1	14	1	2
**Thrombocytopenia**	3	1	0	1	0	1	8	1	1
**Anemia**	23	2	0	14	1	0	29	4	0
**Hemorrhage**	2	0	0	0	0	0	1	0	0
**Infection**	7	0	0	2	0	0	3	1	0
**Fever (No Infection)**	11	0	0	7	0	0	14	0	0
**GU**	1	0	0	1	0	0	5	1	0
**Nausea**	17	0	0	10	1	0	16	3	0
**Vomiting**	6	0	0	3	1	0	7	2	1
**Diarrhea**	7	1	0	4	0	1	7	0	2
**Stomatitis**	3	0	0	2	0	0	4	0	0
**Liver**	21	8	1	18	5	1	24	6	2
**Pulmonary**	1	0	0	0	1	0	0	1	0
**Cardiac**	0	1	0	1	0	0	1	1	0
**Skin**	5	1	0	1	0	0	3	0	0
**Phlebitis**	0	0	0	0	1	0	1	0	0
**Local (No Phlebitis)**	0	0	0	0	0	0	1	0	0
**Alopecia**	0	0	0	0	0	0	4	0	0
**Weight Gain**	4	0	0	3	0	0	2	0	0
**Weight Loss**	8	0	0	7	0	0	6	0	0
**Neuro-sensory**	1	0	0	1	0	0	1	1	0
**Neuro-motor**	4	1	0	3	0	0	2	3	0
**Neuro-psych**	1	0	0	0	0	0	0	0	0
**Neuro-clinical**	2	0	0	4	0	0	7	1	0
**Metabolic**	2	0	0	2	0	0	8	0	0
**Abdominal cramps**	0	0	0	0	0	0	1	0	0
**Bad taste**	0	0	0	1	0	0	0	0	0
**Anorexia**	6	0	0	3	0	0	2	0	0
**Epigastric distress**	1	0	0	0	0	0	1	0	0
**Insomnia**	1	0	0	0	0	0	1	0	0
**Edema**	1	0	0	3	0	0	2	0	0
**Arthralgia**	0	0	0	1	0	0	1	0	0
**Myalgia**	3	0	0	0	0	0	3	0	0
**Fatigue**	18	2	0	10	2	0	14	0	0
**Hiccups**	1	0	0	0	0	0	0	0	0
**Lightheadedness**	1	0	0	0	0	0	0	0	0
**Sweats**	1	0	0	0	1	0	1	0	0
**Bone pain**	1	0	0	0	0	0	0	0	0
**Flu-like symptoms**	3	1	0	3	1	0	2	0	0
**Chills**	1	0	0	2	0	0	0	0	0
**Granulocytopenia**	6	0	1	5	0	1	6	1	3
**Sinusitis**	1	0	0	0	0	0	0	0	0
**Abdominal distention**	1	0	0	0	0	0	1	0	0
**Ileus**	0	0	0	0	0	0	0	2	0
**Dysuria**	0	0	0	0	0	0	1	0	0
**Pain, other**	0	0	0	0	1	0	1	0	0
**All other toxicities**	0	1	0	1	0	0	0	1	0

**Table 1.** Overall response rate based on final strata criteria (*n* = 87).

	Total	Stratum 1	Stratum 2	Stratum 3
Response	*N* (%)	*N* (%)	*N* (%)	*N* (%)
**Complete response**	1 (1 .2)	0 (0)	0 (0)	1 (3.3)
**Partial response**	2 (2.3)	1 (3.0)	1 (4.2)	0 (0.0)
**Stable disease**	32 (36.8)	14 (42.4)	9 (37.5)	9 (30.0)
**Progressive disease**	48 (55.2)	17 (51 .5)	13 (54.2)	18 (60.0)
**Not evaluable**	4 (4.6)	1 (3.0)	1 (4.2)	2 (6.7)

**Table 2.** Overall response rate based on initial strata criteria (*n* = 100).

	Total	Stratum 1	Stratum 2	Stratum 3
Response	*N* (%)	*N* (%)	*N* (%)	*N* (%)
**Complete response**	1 (1.0)	0 (0.0)	0 (0.0)	1 (2.5)
**Partial response**	2 (2.0)	1 (2.9)	1 (3.9)	0 (0.0)
**No change/ stable**	36 (36.0)	14 (41.2)	11 (42.3)	11 (27.5)
**Progressed**	57 (57.0)	18 (52.9)	13 (50.0)	26 (65.0)
**Unevaluable**	4 (4.0)	1 (2.9)	1 (3.9)	2 (5.0)

**Table 3.** Survival data based on initial strata criteria (*n* = 100).

	Overall survival (months) median (95% Cl)	Progression free survival (months) median (95% Cl)
**Total**	10.5 (7.3, 14.6)	1 .8 (1 .5, 2.6)
**Stratum 1**	14.5 (8.0, 19.9)	2.1 (1 .4, 2.7)
**Stratum 2**	12.5 (5.0, 17.0)	2.6 (1 .4, 3.5)
**Stratum 3**	7.3 (4.9, 14.3)	1.7 (1 .4, 2.3)

**Table 4.** Response by thymidylate synthase staining.

	Complete response	Partial response	Stable disease	Progressive disease	Unevaluable
**Low (0, 1+)**	0	0	4	4	0
**High (2+, 3+)**	0	1	16	25	1
**Total**	0	1	20	29	1

**Figure 1.**
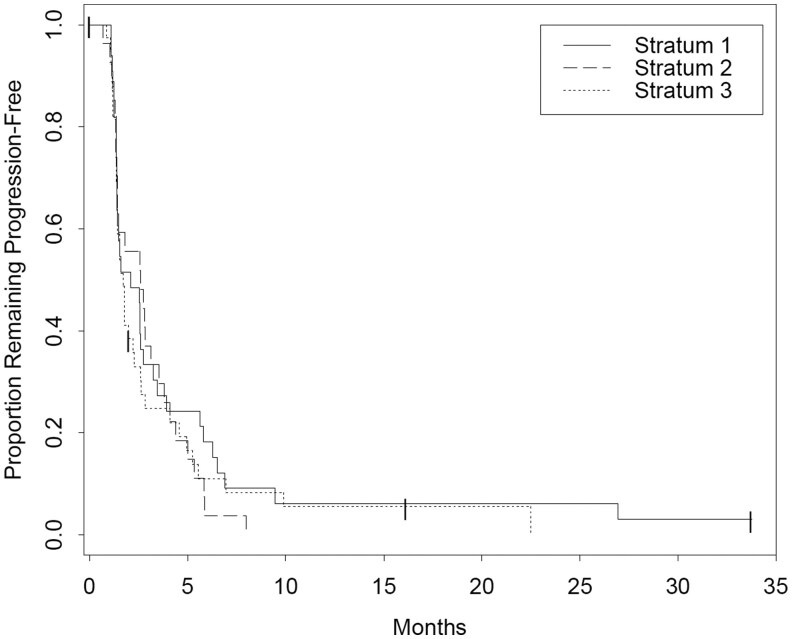
Progress-free survival of entire population as defined by original strata.

**Figure 2.**
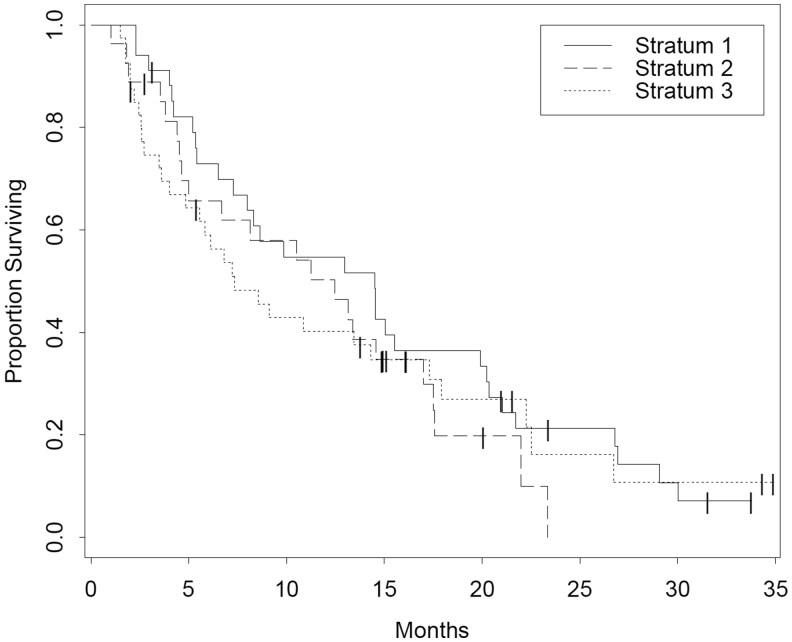
Overall survival of entire population as defined by original strata.

## Discussion

Antimetabolite therapies represent cornerstones in the treatment of cancers. Specifically, inhibition of one carbon metabolism through folate cofactors form the backbone of multiple cancer therapeutics.^1^ Among these targets, inhibition of TS by 5-FU is an important therapeutic mechanism.^2^ 5-FU, however, is not a direct inhibitor of TS, instead forming a ternary complex as activated fluorodeoxyuridylate to interfere with thymidylate production and ultimately DNA synthesis.^3^ In contrast, raltitrexed was developed as a direct inhibitor of TS, with the goal of decreased toxicity through more specific inhibition. Raltitrexed is transported intracellularly through the reduced folate carrier protein. Intracellularly, raltitrexed undergoes polyglutamation prior to inhibiting the binding of TS to folate.^4^ Similar to 5-FU, the end result is decrease in DNA synthesis and repair leading to cytotoxicity.

Given differing inhibitory pathways of 5-FU and raltitrexed, this trial was conducted to evaluate activity of raltitrexed in CRC, in particular its efficacy after exposure to 5-FU and leucovorin. *In vitro* analysis has shown leucovorin can compete with raltitrexed in terms of intracellular transport and polyglutamation.^5^ Prior to the initiation of this study, a phase I study showed limited responses to raltitrexed after exposure to 5-FU regimens with leucovorin, possibly indicating that prior exposure to 5-FU or leucovorin could lead to resistance to raltitrexed.^6^

To examine activity of raltitrexed in treatment naïve and post 5-FU/leucovorin CRC patients, this trial initially enrolled patients both with prior 5-FU/leucovorin exposure and treatment naïve patients. This study also evaluated the role of TS expression to response to raltitrexed. Prior studies have shown that TS is increased with exposure to 5-FU. Because raltitrexed is a direct inhibitor of TS, it was hypothesized that levels of TS expression may serve as a predictive marker of response. As a result, the study incorporated IHC staining of TS of tumor samples for enrolled patients.

This study did not show significant activity of raltitrexed in either the untreated or 5-FU/leucovorin treated population in colorectal cancer, with or without leucovorin. The low ORR compared to historical single agent 5-FU/leucovorin (up to 20%) in untreated patients may suggest that non-TS dependent mechanisms play important therapeutic roles in CRC.^7^ However, the ORR compared to other trials with raltitrexed was lower than previously reported for CRC patients in the first-line setting (3% vs ∼19%).^4^ The median OS across all strata was comparable to prior studies (10.5 months vs 9.7-10.9 months). Toxicity was also in line with prior reports with leukopenia, diarrhea, and elevated transaminases as most common adverse events seen with raltitrexed. Although raltitrexed combinations with oxaliplatin or irinotecan have been studied in clinical trials, its safety and efficacy were similar to 5-FU and these combination treatments are not widely used clinically.^4^

The benefit of raltitrexed after 5-FU progression was not shown in this trial, suggesting that specific TS inhibition is not enough to overcome resistance. In current clinical practice, raltitrexed has been used in the setting of 5-FU toxicity. In particular, cardiotoxicity with 5-FU due to coronary vasospasm is a well reported toxicity.^8^^,^^9^ Although bolus 5-FU may be used in this setting, the frequency of dosing (weekly or daily for 5 days) may be less advantageous compared to raltitrexed (every 3 weeks). In addition, unlike 5-FU which causes significant toxicity in the presence of dihydropyrimidine dehydrogenase (DPD) deficiency, raltitrexed can be metabolized regardless of DPD expression. Within the literature, there have been reports of raltitrexed being used in this setting, however prospective data are lacking and likely difficult to obtain given the rarity of these specific clinical situations.^10^^,^^11^

Although direct targeting of TS in this case did not seem to be beneficial once resistance to 5-FU/leucovorin occurred, targeting of antifolate metabolism remains a key pathway in gastrointestinal cancers. Since this trial was reported, trifluridine/tipiracil has been approved in treatment of CRC after resistance to 5-FU-based regimens.^12^^,^^13^ Trifluridine/tipiracil inhibits TS but also leads to DNA damage through its incorporation to DNA strands after undergoing intracellular phosphorylation.^14^ Despite low ORR in phase III trials, there was an overall survival benefit compared to placebo, with increased benefit in combination with bevacizumab.^12^ Whether treatment with raltitrexed in this setting may improve survival outcomes despite low ORRs has not been studied. However, given the small improvements in overall survival with trifluridine/tipiracil, it is clear that novel therapeutics in CRC are needed.

More recently, the methionine salvage pathway, adjacent to the folate pathway, has been of interest in the setting of deletion of the MTAP gene.^15^ Synthetic lethality has been proposed with novel MAT2A and PRMT5 inhibitors.^16^ MTAP is also a key enzyme in purine biosynthesis; preliminary evidence indicates that non-small cell lung cancer and urothelial carcinoma with MTAP deletions may be more sensitive to antifolate drug pemetrexed.^17^ In gastrointestinal cancers, whether this pathway may still play a role after prior exposure to folate alteration through 5-FU treatment will be an important question. This trial suggested that prior targeting of folate pathways may predispose to resistance to further inhibition of this pathway. However, despite widespread 5-FU utilization in gastrointestinal cancers, biomarkers of response and resistance that are clinically useful are still lacking. IHC staining for TS was used in this trial as a potential predictive biomarker. However, the response rates in each of the strata were too low to make conclusions on level of TS expression as a predictive marker for raltitrexed response. While high TS has been associated with decreased response to raltitrexed and 5-FU, it has not been shown to be a reliable biomarker in 5-FU responsiveness and has not been adopted clinically.^18–20^ As novel therapeutics are developed targeting antifolate and broader antimetabolite pathways, evaluation of potential biomarkers will be especially important in gastrointestinal cancers given the propensity for first-line regimens to include 5-FU.

## Data Availability

The data from the present publication will be made available by request from the NCTN/NCORP Data Archive: https://nctn-data-archive.nci.nih.gov
